# Selective Inhibitors of Histone Deacetylases 1 and 2 Synergize with Azacitidine in Acute Myeloid Leukemia

**DOI:** 10.1371/journal.pone.0169128

**Published:** 2017-01-06

**Authors:** Chengyin Min, Nathan Moore, Jeffrey R. Shearstone, Steven N. Quayle, Pengyu Huang, John H. van Duzer, Matthew B. Jarpe, Simon S. Jones, Min Yang

**Affiliations:** Acetylon Pharmaceuticals Inc., Boston, Massachusetts, United States of America; European Institute of Oncology, ITALY

## Abstract

Acute myeloid leukemia (AML) is a heterogeneous group of hematopoietic stem cell disorders characterized by defects in myeloid differentiation and increased proliferation of neoplastic hematopoietic precursor cells. Outcomes for patients with AML remain poor, highlighting the need for novel treatment options. Aberrant epigenetic regulation plays an important role in the pathogenesis of AML, and inhibitors of DNA methyltransferase or histone deacetylase (HDAC) enzymes have exhibited activity in preclinical AML models. Combination studies with HDAC inhibitors plus DNA methyltransferase inhibitors have potential beneficial clinical activity in AML, however the toxicity profiles of non-selective HDAC inhibitors in the combination setting limit their clinical utility. In this work, we describe the preclinical development of selective inhibitors of HDAC1 and HDAC2, which are hypothesized to have improved safety profiles, for combination therapy in AML. We demonstrate that selective inhibition of HDAC1 and HDAC2 is sufficient to achieve efficacy both as a single agent and in combination with azacitidine in preclinical models of AML, including established AML cell lines, primary leukemia cells from AML patient bone marrow samples and *in vivo* xenograft models of human AML. Gene expression profiling of AML cells treated with either an HDAC1/2 inhibitor, azacitidine, or the combination of both have identified a list of genes involved in transcription and cell cycle regulation as potential mediators of the combinatorial effects of HDAC1/2 inhibition with azacitidine. Together, these findings support the clinical evaluation of selective HDAC1/2 inhibitors in combination with azacitidine in AML patients.

## Introduction

AML is the most prevalent acute leukemia in adults and results from the transformation of primitive hematopoietic stem and progenitor cells, leading to increased proliferation and impaired differentiation of immature myeloid progenitors [1]. Treatment options for AML patients are limited and outcomes are poor [2]. There is a high unmet medical need in these patients for novel treatment options. Considering the high intrinsic genetic instability and heterogeneity of AML, it is generally believed that combination regimens will be necessary to achieve the desired clinical efficacy. Recent discoveries have highlighted an important role of dysregulated epigenetic mechanisms in the pathogenesis of AML [3–5]. Epigenetic changes include DNA modifications, such as cytosine methylation, modifications of histone proteins, such as histone acetylation and histone methylation, and RNA-associated gene silencing. Development of DNA methyltransferase inhibitors has been the most successful in this disease. VIDAZA^®^ (azacitidine), which has demonstrated efficacy in AML preclinical models [6–11] and clinical trials [12, 13], was recently approved by the European Medicines Agency for the treatment of elderly patients with AML. HDAC inhibitors are another class of epigenetic therapies which are under extensive development for AML and other hematologic malignancies. In multiple preclinical models of AML, HDAC inhibitors such as panobinostat, vorinostat, or entinostat, have demonstrated antitumor activities either as a single agent or in combination settings through induction of differentiation, cell cycle arrest and/or apoptosis [14–27]. HDAC inhibitors have also shown promising clinical activity in combination with agents with known anti-leukemia activity, including DNA methyltransferase inhibitors and chemotherapies, in AML patients [2, 28–36]. However, adding non-selective HDAC inhibitors to combination regimens often results in increased toxicities which can lead to dose reduction and early treatment discontinuation [33, 36–45]. Therefore, isozyme-selective HDAC inhibitors with improved safety profiles may overcome this hurdle and provide additional clinical benefit to patients.

In humans there are 11 classical HDAC isoforms [46]. HDACs 1–3 are enzymatically active members of transcriptional corepressor complexes, responsible for chromosomal compaction and gene repression through removing acetyl groups from lysine residues on histones. Initial genetic dissection of the role of specific HDACs in murine models has revealed that HDAC1 and HDAC2 play redundant and essential roles in tumor cell growth *in vitro* and *in vivo* [47, 48]. Furthermore, co-inhibition of HDAC1 with HDAC2 by genetic and pharmacological approaches was shown to mediate robust pro-apoptotic responses in models of lymphoma and B-cell acute lymphoblastic leukemia [22, 23, 47, 49]. Together, these findings suggest that pharmacological inhibition of HDAC1 and HDAC2 is sufficient for anti-tumor activities in AML.

Here, we describe the preclinical development of series of oral and selective inhibitors of HDAC1 and HDAC2 based upon the biaryl aminobenzamide scaffold [50–52]. Our results demonstrate potent anti-leukemic activities of HDAC1/2-selective inhibitors both as single agents and in combination with azacitidine in multiple *in vitro*, *ex vivo* and *in vivo* preclinical models of AML.

## Materials and Methods

### Cell culture

MV-4-11, Kasumi-1, and HL-60 AML cell lines were all obtained from ATCC. MOLM-13 and NB-4 cell lines were obtained from DSMZ. All cell lines were cultured in Roswell Park Memorial Institute (RPMI) 1640, supplemented with 10% (MV-4-11, MOLM-13 and NB-4) or 20% FBS (Kasumi-1 and HL-60) and 100 U/mL penicillin and 100 μg/mL streptomycin. The identity of each cell line was validated and documented by the suppliers. All experiments were performed with cells maintained at low passage numbers.

### HDAC enzyme assays

*In vitro* biochemical assays were performed as described previously [52, 53]. Specifically, compounds were dissolved and diluted in assay buffer (50 mM HEPES, pH 7.4, 100 mM KCl, 0.001% Tween-20, 0.05% BSA, and 20 μM Tris (2-carboxyethyl) phosphine) to 6-fold the final concentration. HDAC enzymes (BPS Biosciences) were diluted to 1.5-fold of the final concentration in assay buffer and pre-incubated with ACY-957 or ACY-1035 for 24 hours at 4°C before the addition of the substrate. The amount of substrate (acetyl-lysine tripeptide) used for each enzyme was equal to the Michaelis constant (Km), as determined by a titration curve. Substrate was diluted in assay buffer to 6-fold of the final concentration with 0.3 μM sequencing grade trypsin (Sigma-Aldrich). The substrate/trypsin mix was added to the enzyme/compound mix and the plate was shaken for 5 seconds and then placed into a SpectraMax M5 microtiter (Molecular Devices) plate reader. The enzymatic reaction was monitored over 30 minutes for release of 7-amino-4-methoxy-coumarin after deacetylation of the lysine side chain in the peptide substrate, and the linear rate of the reaction was calculated.

### Cell viability assays

Single agent and combination assays were carried out in 384-well plates by plating 2.5 x 10^5^ cells/mL in 30 μL media/well. Compounds and/or solvent dimethyl sulfoxide (DMSO) were added at the same time using an HP D300 digital dispenser (Hewlett Packard) in triplicate or quadruplicate and incubated for 72 hours at 37°C. Viability was measured by adding 25 μL CellTiter-Glo (Promega), incubating for 10 minutes, and measuring luminescence on a SpectraMax (Molecular Devices). For single agent treatment, 11 doses at a range of 0.1 μM to 100 μM were tested for each drug and IC_50_ values calculated using GraphPad Prism 6. For combination treatment, cells were treated in quadruplicate with 7 concentrations of HDAC inhibitors plus 7 concentrations of azacitidine with the ratio of the drug doses staying at approximately IC_50_: IC_50_. Combination index (CI) was calculated using Calcusyn software (Biosoft, UK) and reported are the average of 3 independent experiments. CI < 1 indicates synergism, CI = 1 indicates additivity, and CI > 1 indicates antagonism.

### Immunoblotting

The EpiQuick Total Histone Extraction kit (Epigentek) was used to isolate histones. Histone lysates were size separated on Bolt 10% Bis-Tris gradient gels (Life Technologies), transferred to polyvinylidene difluoride membranes, blocked in 5% BSA, and detected using standard western blotting techniques. Antibodies used for western blotting: anti-acetylated histone H3 lysine 56 (H3K56ac) (Abcam, catalog 76307), anti-acetylated histone H2B lysine 5 (H2BK5ac) (Cell Signaling Technology, catalog 2574), and anti-total-histone H4 (EMD Millipore, catalog 05-858).

### Flow cytometry

Single agent and combination assays were carried out in 6-well format by plating 2.5x10^5^ cells/mL in 5 mL of culture media. All compounds and/or solvent DMSO were added concurrently at the indicated doses. Flow cytometry was performed to measure surface level of CD11b, cell cycle distribution, and induction of apoptosis. CD11b and apoptosis were analyzed on a FC500 flow cytometer (Becton Dickenson) and cell cycle profiled on an Attune NxT (Life Technologies).

For CD11b analysis, 72 hours after treatment, 1.0 x 10^6^ cells for each condition were washed once in wash buffer (PBS with 0.1% BSA), stained in wash buffer on ice for 1 hour with PE-anti-human CD11b (Biolegend, catalog 301306) or PE-mouse IgG1κ (BD Pharmingen catalog 554680), and then washed 3 times before flow cytometry analysis. The frequency of CD11b positive cells out of total viable cells identified by forward and side scatter profile was quantified.

For cell cycle analysis, 72 hours after treatment, 1 mL of cells from each condition were incubated with EdU for 1 hour and then mixed with detection cocktail according to the manufacturer’s protocol (Life Technologies, ClickiT Plus EdU 488 Assay). EdU labeled cells were then mixed with FxCycle Far Red Stain (Life Technologies) and 100 μg RNase A cocktail (Life technologies) for 30 minutes before flow cytometry analysis. Edu positive populations representing S phase cells were quantified.

For apoptosis analysis, 96 hours after treatment, 1 mL of cells from each condition were stained using the 488-Annexin V Dead cell kit according to the manufacturer’s protocol (Life Technologies, catalog V13245). Annexin V-positive populations were quantified.

### Primary bone marrow sample proliferation assay

Fresh bone marrow samples from AML patients were plated into 96-well plates containing ACY-957 alone, azacitidine alone or the combination and incubated for 96 hours. AML blasts were characterized according to their light scatter properties and stained with antibodies to the following markers: CD11b, CD45, CD13, CD34, CD64, CD117 and HLA-DR as described previously [54, 55]. AML blasts were incubated in RPMI 1640 medium (20% FBS, 2% HEPES, 1% penicillin-streptomycin, and 1% L-glutamine) containing a human cytokine cocktail to stimulate proliferation of leukemic cells. The cytokine cocktail included 0.1 ng/μL SCF (StemCell Technologies, catalog 02630), 0.05 ng/μL IL-3 (StemCell Technologies, catalog 02503), 0.04 ng/μL IL-6 (Miltenyi Biotech, catalog 130-095-365), 0.2 ng/μL GM-CSF (Peprotech, catalog 300–03), 0.2 ng/μL G-CSF (Peprotech, catalog 300–23), 0.004 U/mL Erithropoietin (StemCell, catalog 02625), 0.94 μg/μL Transferrin (Sigma Aldrich, catalog T8158), 0.1 ng/μL 2-Mercaptoethanol (Sigma Aldrich, catalog M7154). To identify live leukemic cells by flow cytometry, two antibodies that unequivocally identify the pathologic cell population in the patient samples were selected in combination with annexin V. Those cells without annexin V staining and with appropriate markers were considered live leukemic cells, as described previously [54, 55]. Proliferation inhibition was measured as the difference in the number of live leukemic cells in a well with drugs versus the vehicle control treated wells.

For single agent treatment, 38 patient samples were tested with an 8-point dose-response of each agent. After 96-hour treatment, live leukemic cells were quantified by flow cytometry and IC_50_ values calculated by GraphPad Prism 6. For combination treatments, 30 patient samples were assessed. Three concentrations of each drug were chosen based on the estimated IC_50_ values, and eight different combination ratios were tested concurrently with each single agent on matched samples. The quantification of live leukemic cells and the calculation of IC_50_ values for single agent were conducted as above. The drug interactions were characterized by computing the standard CI as determined by the method of Chou-Talalay [56]. CI values below 1 indicate synergism.

Studies were performed at Vivia Biotech, Madrid, Spain. Studies were approved by the following ethics committees: Clinical Research Ethics Committee of the Hospital Universitario Doce de Octubre, Madrid; Clinical Research Ethics Committee from Cáceres; Clinical Research Ethics Committee of the Hospital Universitario Virgen Macarena, Sevilla; Biomedical Research Ethics Committee Hospital Universitario y Politécnico La Fe, Valencia; Clinical Research Ethics Committee of the Hospital Universitario de Salamanca; Clinical Research Ethics Committee from Galicia; Ethics Committee of Clinical Research-Committee for the Ethics in Research of the Hospital Universitario de Gran Canaria Doctor Negrin; Clinical Research Ethics Committee of the Hospital Clinico Universitario de Valencia; Clinical Research Ethics Committee of the Hospital Universitario Germans Trias i Pujol, Barcelona; Clinical Research Ethics Committee of the Grupo Hospital de Madrid; Clinical Research Ethics Committee of the Hospital General Universitario Gregorio Maraňón, Madrid; Clinical Research Ethics Committee of the Hospital Universitario Ramón y Cajal, Madrid. All the participants provided their written informed consent to participate in this study.

### Primary bone marrow sample colony formation assay

Frozen bone marrow samples from AML patients were treated with ACY-957 alone, azacitidine alone or the combination. Specifically, compounds were dissolved in DMSO and added to tubes of methylcellulose-based medium (Reachbio, catalog 1103) containing cytokine formulations to support colony growth of AML blasts. Frozen bone marrow samples were thawed and approximately 4 x 10^4^ cells/culture were plated in triplicate for each treatment condition. The dishes were cultured at 37°C for a total of 14 days, after which the resultant colonies were evaluated and enumerated based on morphology.

For single agent efficacy, 7 AML samples were tested with a 6-point dose-response of each agent. IC_50_ values were generated by GraphPad Prism 6. The 5 samples that supported the growth of sufficient numbers of colonies were further tested with combination treatment. For each sample, one dose of each drug was chosen based on the following criteria: inhibits colony formation no more than 50%; gives similar levels of inhibition as each other.

Studies were performed at Reachbio LLC, Seattle, USA. Studies were approved by Western Institutional Review Board and Alpha Institutional Review Board. All the participants provided their written informed consent to participate in this study.

### *In vivo* model of AML

Female Crl:NU(NCr)-*Foxn1*^*nu*^ mice were injected subcutaneously with 1 x 10^7^ MOLM-13 cells in 50% Matrigel in the right flank. When tumors reached an average size of 100–150 mm^3^, mice (n = 10/group) were randomized into 4 treatment groups: azacitidine, ACY-957, azacitidine plus ACY-957 and vehicle control. Azacitidine was administered intravenously (i.v.) twice weekly at 5.5 mg/kg in saline while ACY-957 was administered orally (p.o.) daily at 125 mg/kg in 0.5% hydroxypropyl methylcellulose in deionized water. Mice were treated for 28 days. The dosing schedule was chosen based on tolerability of the treatment as well as pharmacokinetic profile of ACY-957 in mice. Tumors were measured twice weekly in two dimensions using calipers and volume was calculated by width^2^ x length/2. Animals were monitored individually for tumor growth until day 34. Each animal was euthanized for tumor progression when its tumor reached the 2000 mm^3^ volume endpoint and time to endpoint (TTE) was determined for each mouse. To calculate TTE, tumor volume data were log transformed and linear regression performed on the data set comprising the first observation that exceeded the endpoint volume and the three consecutive observations that immediately preceded the attainment of the endpoint volume. TTE for each mouse was calculated with the following equation: TTE = log_10_ [(endpoint volume)–intercept]/slope of linear regression. In instances in which the log-transformed calculated TTE preceded the day prior to reaching endpoint or exceeded the day of reaching tumor volume endpoint, a linear interpolation was performed to approximate TTE. Any animal determined to have died from treatment-related causes was assigned a TTE value equal to the day of death. Any animal that died from non-treatment-related causes was excluded from the analysis. Any animal that did not reach endpoint was euthanized at the end of the study and assigned a TTE equal to the last study day (Day 34). Survival curve was plotted using GraphPad Prism 6 and statistical analysis performed by log-rank (Mantel-Cox) test.

All animal work was performed at Charles River Discovery Services, Morrisville, NC (CR DS-NC). The identity of MOLM-13 cell line was validated and documented by Charles River Discovery Services. The study was approved by the CR DS-NC Institutional Animal Care and Use Committee. This site has an approved Assurance Statement (A4358-01) on file with the Office of Laboratory Animal Welfare (OLAW), National Institutes of Health (NIH) and is accredited by the Association for Assessment and Accreditation of Laboratory Animal Care International (AAALAC International). Animals were obtained from an approved vendor and were housed under conditions which met the requirements specified in the *Guide for the Care and Use of Laboratory Animals* from the National Research Council. The health and welfare of all animals was assessed on a daily basis and animals observed with abnormal clinical signs were brought the attention of the veterinary staff for monitoring and recommendation of appropriate treatment, including supportive care. Based on veterinary recommendation, animals experiencing severe or chronic pain or distress that could not be relieved were humanely euthanized. Animals on this study were euthanized by exposure to an overdose of isoflurane consistent with the AVMA Guidelines of Euthanasia for the Euthanasia of Animals: 2013 Edition. There were no unexpected deaths in this study.

IACUC approved general humane end points for tumor models that were applied to this study included: 1) Tumor volume reaches 2000 mm^3^, 2) Any individual animal with a single observation of more than 30% body weight loss or three consecutive measurements of more than 25% body weight loss, 3) Any group with a mean body weight loss of more than 20% or more than 10% mortality will stop dosing. The group is not euthanized and recovery is allowed. Within a group with more than 20% weight loss, individuals hitting the individual body weight loss endpoint will be euthanized. If the group treatment related body weight loss is recovered to within 10% of the original weights, dosing may resume at a lower dose or less frequent dosing schedule. Exceptions to non-treatment body weight % recovery may be allowed on a case-by-case basis based on veterinary approval in consultation with the Study Director.

### Gene expression microarray analysis

MV-4-11 cells were treated for 48 hours with vehicle DMSO, 1 μM ACY-1035, 1 μM azacitidine or the combination of 1 μM azacitidine plus 1 μM ACY-1035. Total RNA from cells was isolated using the RNeasy^®^ Mini Kit. Quality of the RNA samples was assessed by 260/280 nm and 260/230 nm absorption spectrum profiles using a NanoDrop spectrophotometer (Thermo Scientific, Waltham, MA).

Gene expression profiling sample preparation, hybridization, and data acquisition was performed by the Covance Genomics Laboratory (Seattle, WA) using total RNA and Affymetrix GeneChip PrimeView^™^ Human Gene Expression Array (Affymetrix, Santa Clara, CA) according to manufacturer’s protocol. Data analysis was performed using BRB-ArrayTools [57]. Raw data CEL files were processed with the robust multi-chip average (RMA) algorithm [58]. This data has been uploaded to the National Center for Biotechnology Information (NCBI) Gene Expression Omnibus (GEO) website with series accession number of GSE84440.

Venn diagram analysis was performed by comparing each treatment condition to vehicle control and then selecting Affymetrix qualifiers up- or down-regulated by at least 2 fold. Gene symbols were used to compute gene list overlaps using Venny written by JC Oliveros (http://bioinfogp.cnb.csic.es/tools/venny). Heatmap analysis was performed by comparing each treatment condition at 48 hours to vehicle control. Affymetrix qualifiers showing additive or synergistic changes in gene expression following the combination treatment were selected using the fold change filters outlined in S1 Table. The heatmap resulting from these lists was generated using matrix2png web interface (http://www.chibi.ubc.ca/matrix2png/bin/matrix2png.cgi) with log base 2 intensity values for each qualifier normalized to have mean value of zero and variance one [59].

Gene set enrichment analysis (GSEA) was performed using the publicly available GSEA software from the Broad Institute. Log base 2 gene expression data was used to query the ‘C3 motif TFT: transcription factor targets’ gene sets within the Molecular Signatures Database collection [60]. Pairwise sample comparisons were made by collapsing the data set to gene symbols based on maximum qualifier signal and then using a weighted difference of means metric for ranking genes. Gene set permutations were performed to generate nominal P-values and false discovery rate (FDR) for each gene set.

### Quantitative real time PCR (qPCR)

RNA isolations were performed on a Qiacube (Qiagen) using the RNeasy Micro Kit (Qiagen) according to manufacturer’s protocol. RNA was converted to cDNA using the High Capacity RNA-to-cDNA Kit (Life Technologies) and qPCR performed using the Taqman Gene Expression Max Mix according to manufacturer’s protocol (Applied Bioscience). The following ThermoFisher taqman probes were used: GATA2 (Hs01060665_g1), GAPDH (Hs0275899_g1), ACTB (Hs00231119_m1), CDKN1A (Hs00355782_m1), CDKN1C (Hs00175938_m1), HES1 (Hs00172878_m1).

### GATA2 overexpression and growth assay

The GATA2 overexpression vector (oeG2) was constructed by removing the GFP cassette of the pLKO.1-puro-CMV-TurboGFP vector (Sigma, Catalog SHC003) and replacing it with the full-length *GATA2* transcript sequence (Refseq NM_001145661) (GenScript, Piscataway, NJ), as described previously [52]. pLKO.1-puro-CMV-TurboGFP vector was used as a control (oeGFP). Cells were transduced, recovered for 48 hours and selected for two days with 1 μg/mL puromycin. Stable cells were then cultured for an additional day in RPMI 1640 media before further use.

For growth assays, stable cells were plated at 1.0 x 10^5^ cells/mL in 200 μL of culture media in 96-well plates. Cell viability was determined by CellTiter-Glo as above on day 0, 2, 3 and 6. At each time point, a reference curve of absolute viable cell numbers was generated by measuring the viability of parental MV-4-11 cells at a range of cell numbers. The viable cell numbers for GATA2 and GFP overexpressing cells were derived from the reference curve.

### Statistical analysis

Statistical analysis of survival curves was performed by log-rank (Mantel-Cox) test using GraphPad Prism 6 and statistical significance assumed at P < 0.05. For statistical analysis of GSEA, gene set permutations were performed to generate nominal P-values and FDR for each gene set and statistical significance assumed at FDR < 0.05. All other statistical analysis was performed by two-tailed Student’s t-test using Excel and statistical significance assumed at P < 0.05.

## Results

### ACY-957 and ACY-1035 selectively inhibit HDAC1 and HDAC2

**Fig 1:**
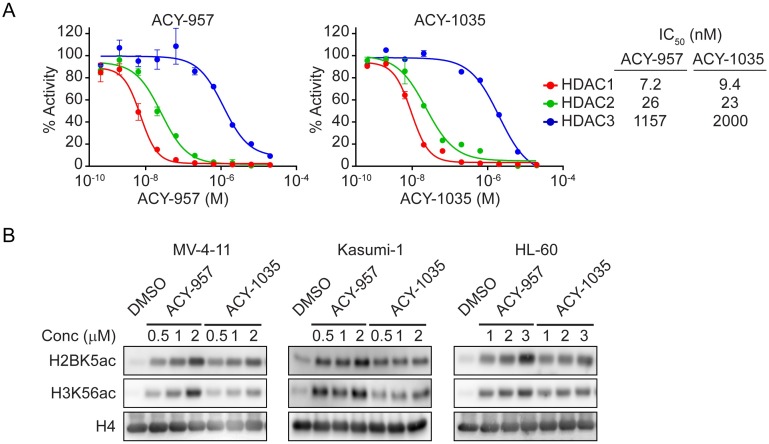
ACY-957 and ACY-1035 selectively inhibits HDAC1 and 2. (A) *In vitro* biochemical assay for HDAC1, HDAC2 or HDAC3 inhibition by ACY-957 (*left panel*) and ACY-1035 (*right panel*). Mean ± SD, n = 3. (B) MV-4-11, Kasumi-1 or HL-60 cells were treated with the indicated doses of compounds for 24 hours and immunoblotting performed for acetylated H2BK5, acetylated H3K56 and total H4. A dose-dependent induction of histone acetylation by ACY-957 or ACY-1035 was observed.

ACY-957 and ACY-1035 belong to the biaryl aminobenzamide class of HDAC inhibitors, which possess an internal cavity binding region shown to provide selectivity for HDAC1 and HDAC2 [50–52]. To confirm their selectivity profiles, ACY-957 and ACY-1035 were tested against HDAC1, 2, or 3 in an *in vitro* biochemical assay. ACY-957 had IC_50_ values of 7.2 nM, 26 nM and 1157 nM against HDAC1, 2 and 3, respectively (Fig 1A, *left panel*). ACY-1035 had IC_50_ values of 9.4 nM, 23 nM and 2000 nM against HDAC1, 2 and 3, respectively (Fig 1A, *right panel*). Therefore, ACY-957 and ACY-1035 are HDAC1/2-selective inhibitors with more than 150-fold selectivity for HDAC1 over HDAC3 and approximately 50-fold selectivity for HDAC2 over HDAC3, values consistent with previous reports [51–53, 61]. In addition, ACY-1035 did not inhibit HDAC4 through 9 at the maximum concentration tested of 20 μM (S1 Fig), consistent with previous reports on ACY-957 [52] and on other compounds in the biaryl aminobenzamide class [53, 61].

To confirm the inhibitory activity of these compounds on HDAC1/2, we evaluated their effects on histone acetylation in AML cells. MV-4-11, Kasumi-1 and HL-60 cell lines were treated with increasing doses of ACY-957 or ACY-1035 for 24 hours and histone acetylation measured by immunoblotting. Treatment with both compounds led to a dose-dependent accumulation of acetylation on histone H2B lysine 5 (H2BK5ac) and histone H3 at lysine 56 (H3K56ac) (Fig 1B and S2 Fig), confirming their cellular inhibition of HDACs.

### HDAC1/2 inhibition suppresses viability of AML cells *in vitro* and primary AML blasts *ex vivo*

**Fig 2:**
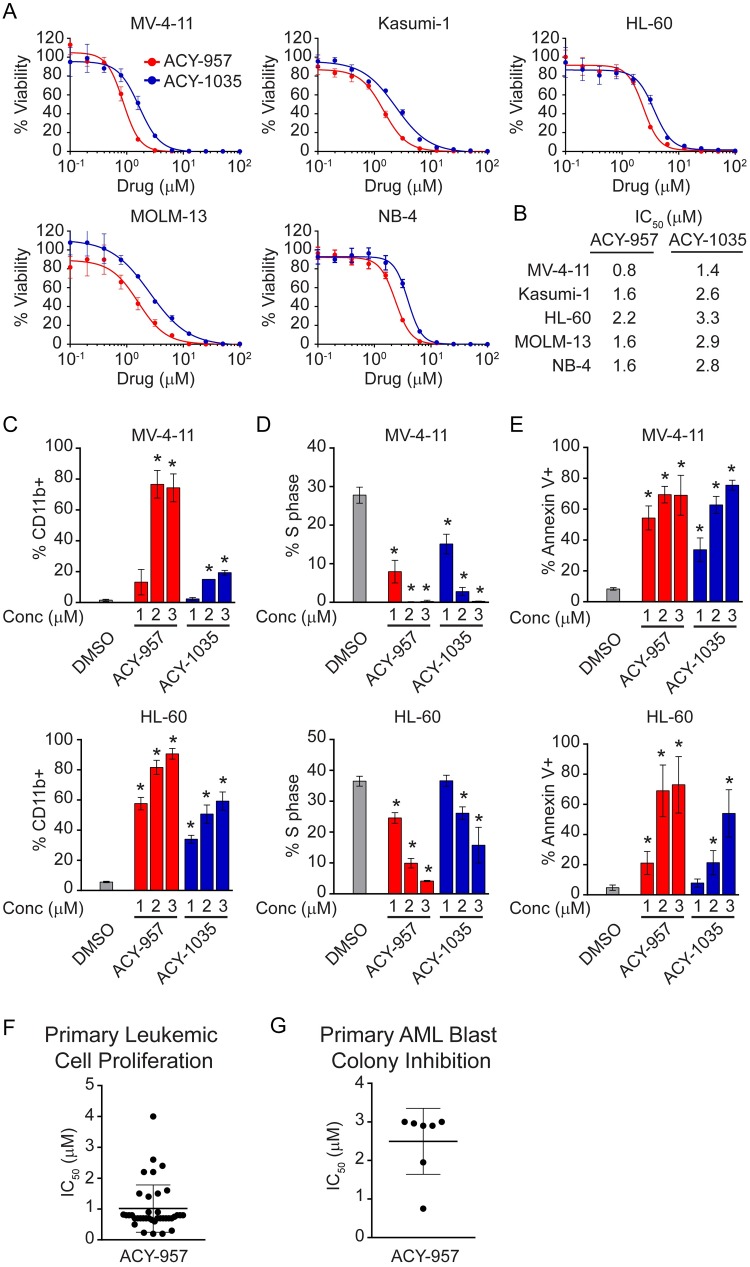
HDAC1/2 inhibition suppresses AML cells *in vitro* and primary AML blasts *ex vivo*. (A) MV-4-11, Kasumi-1, HL-60, MOLM-13 and NB-4 cells were treated with increasing doses of ACY-957 or ACY-1035. After 72 hours, cell viability was measured and response curves plotted using GraphPad Prism 6. Mean ± SD, n ≥ 3. (B) IC_50_ values from curves in ‘A’ were calculated and listed. ACY-957 and ACY-1035 reduced viability of AML cells with an average IC_50_ value of 1.5 μM for ACY-957 and 2.6 μM for ACY-1035, respectively. (C) MV-4-11 (*upper panel*) and HL-60 (*lower panel*) cells were treated with increasing doses of ACY-957 or ACY-1035 for 72 hours. Surface levels of CD11b were measured by flow cytometry and percentage of CD11b positive cells plotted. ACY-957 and ACY-1035 increased the percentage of CD11b positive cells in a dose-dependent manner. Mean ± SD, n = 3 independent experiments. (D) Cells were treated as above. Cell cycle was analyzed by flow cytometry Edu positive population corresponding to S phase cells were quantified and percentage of S phase cells plotted. ACY-957 and ACY-1035 reduced S phase cells in a dose-dependent manner. Mean ± SD, n = 3 independent experiments. (E) Cells were treated at the indicated doses of compounds for 96 hours. Annexin-V positive cells were analyzed by flow cytometry. ACY-957 and ACY-1035 induced apoptosis in a dose-dependent manner. Mean ± SD, n = 3 independent experiments. (F) Fresh primary AML patient samples were plated into 96-well plates containing ACY-957 at 8 doses and incubated at 37°C for 96 hours. Leukemic cells were identified by surface markers as described in the Materials and Methods and viable leukemic cells corresponding to annexin V negative populations quantified by flow cytometry. IC_50_ values were calculated using GraphPad Prism 6 and plotted. ACY-957 inhibited the proliferation of primary leukemic cells with a mean IC_50_ value of 1.1 μM. Bars represent mean ± SD, n = 38. (G) Frozen primary AML patient samples were cultured in methylcellulose-based medium containing cytokines and 6 concentrations of ACY-957 for 14 days as described in the Materials and Methods. Colonies were quantified and IC_50_ values plotted. ACY-957 inhibited colony growth at a mean IC_50_ value of 2.6 μM. Bars represent mean ± SD, n = 7. For all panels, statistical analysis was performed by two-tailed Student’s t-test. * indicates P < 0.05 when comparing compound-treated group to DMSO control.

The effect of ACY-957 and ACY-1035 on viability was assessed in five human AML cell lines representing multiple AML subtypes and common genetic aberrations (Fig 2A and S3 Fig). Both ACY-957 and ACY-1035 potently inhibited cell viability with IC_50_ values in the range of 0.8–3.3 μM, consistent with their demonstrated inhibition of HDAC1/2 at these concentrations (Fig 2B).

We next explored additional anti-leukemic mechanisms of ACY-957 and ACY-1035 using AML cell lines MV-4-11 and HL-60. Both ACY-957 and ACY-1035 led to a dose-dependent induction of differentiation in AML cells 72-hour post-treatment as evidenced by increased surface expression of CD11b (Fig 2C). Both agents also caused a dose-dependent reduction of cells in S phase, consistent with suppression of proliferation (Fig 2D). Furthermore, cells treated for 96 hours exhibited increased staining for annexin V, indicating an induction of cell apoptosis by ACY-957 or ACY-1035 (Fig 2E). Taken together, these data demonstrate that HDAC1/2 inhibition is sufficient to induce differentiation, cell cycle arrest and apoptosis in AML cell lines.

Next, the effects of HDAC1/2 inhibition on primary bone marrow samples from AML patients were assessed in two- and three-dimensional (2D and 3D) *ex vivo* assays. Fresh bone marrow samples from 38 AML patients were treated with increasing doses of ACY-957 in the presence of a cytokine cocktail to stimulate blast proliferation in 2D culture. After 96 hours, live versus apoptotic leukemic cells were detected by annexin V using flow cytometry. ACY-957 inhibited proliferation of leukemic cells from AML patients with a mean IC_50_ value of 1.1 μM (Fig 2F and S4 Fig). Moreover, frozen bone marrow samples from seven AML patients were treated with increasing doses of ACY-957 in methylcellulose-based media containing cytokine formulations to support growth of blast progenitors in the 3D assay. After 14 days in culture, colony numbers were quantified and IC_50_ calculated. ACY-957 inhibited colony growth by AML blasts with a mean IC_50_ value of 2.5 μM (Fig 2G and S4 Fig). These data demonstrate ACY-957 inhibited the growth of primary AML blasts derived from patients.

### HDAC1/2 inhibitors synergize with azacitidine to inhibit transformed phenotypes of AML cell lines and primary AML blasts

**Fig 3:**
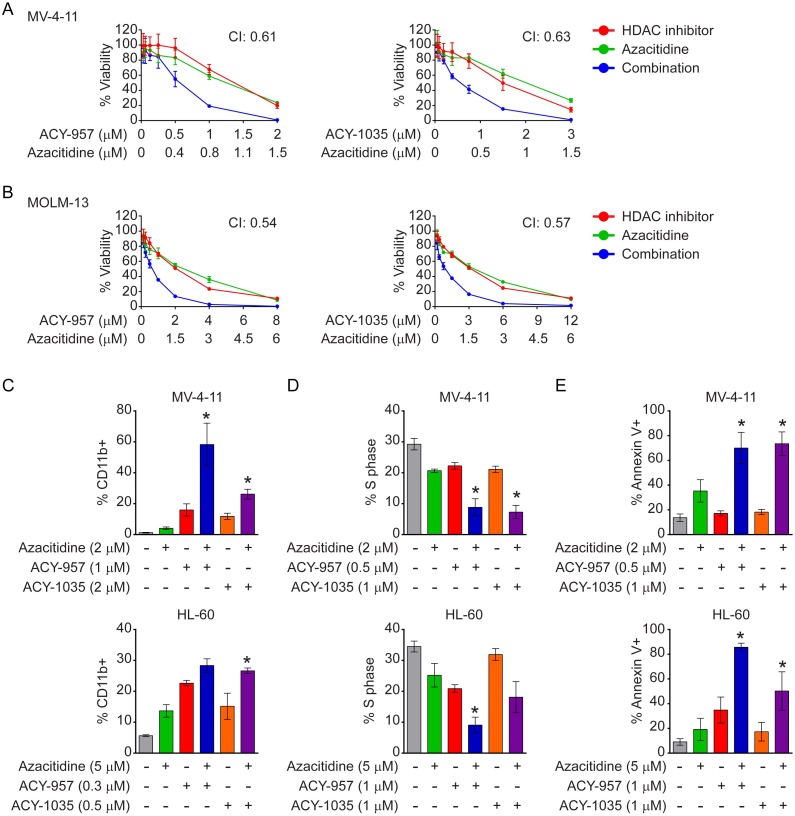
HDAC1/2 inhibition synergizes with azacitidine to inhibit transformed phenotypes of AML cell lines. (A–B) MV-4-11 and MOLM-13 cells were treated with 7 doses of ACY-957 plus azacitidine (*left panels*) or 7 doses of ACY-1035 plus azacitidine (*right panels*) with the ratio of the drug doses staying at approximately IC_50_: IC_50_ as described in the Materials and Methods. Viability was measured and CI values calculated and reported as the average of 3 independent experiments. (C) MV-4-11 (*upper panel*) and HL-60 (*lower panel*) cells were treated with azacitidine, ACY-957 or ACY-1035 either as single agents or in combinations at the indicated doses for 72 hours. Surface levels of CD11b were analyzed as in Fig 2C. Combination treatment of ACY-957 plus azacitidine or ACY-1035 plus azacitidine further increased percentage of CD11b positive cells compared to each single agent. Mean ± SD, n = 3 independent experiments. (D) Cells were treated as in ‘C’ and percentage of S phase cells analyzed and plotted as in Fig 2D. Combination treatment further reduced the percentage of S phase cells compared to single agent treatments. Mean ± SD, n = 3 independent experiments. (E) Cells were treated as above for 96 hours and annexin V positive cells quantified as in Fig 2E. Combination treatment further increased apoptotic cells compared to single agent treatments. Mean ± SD, n = 3 independent experiments. For all panels, statistical analysis was performed by two-tailed Student’s t-test. * indicates P < 0.05 for all 3 comparisons: combination treatment versus DMSO; combination treatment versus azacitidine alone; combination treatment versus HDAC1/2 inhibitor alone.

Gene silencing often involves modification of histone tails and methylation of cytosines in the gene promoter region [62, 63]. HDAC inhibitors can synergize with demethylating agents to relieve transcriptional repression of tumor suppressor genes in cancer [64]. Therefore, the effect of HDAC1/2 inhibitors plus azacitidine, a demethylating agent approved for AML, was assessed on AML cells. The combinatorial effect of azacitidine with ACY-957 or ACY-1035 was first evaluated in viability assays with the two drugs dosed at a constant ratio in MV-4-11 and MOLM-13 cells. A synergistic interaction between azacitidine and ACY-957 or ACY-1035 was observed with average combination index (CI) values below 1 (Fig 3A and 3B). Azacitidine was previously shown to induce differentiation, cell cycle arrest and apoptosis in AML cells [7], similar to our findings with single agent treatment of HDAC1/2 inhibitors shown above. Therefore, the combinatorial effect of azacitidine plus HDAC1/2 inhibitors on differentiation, cell cycle distribution, and apoptosis was evaluated in MV-4-11 and HL-60 cells. Azacitidine in combination with either ACY-957 or ACY-1035 significantly increased the percentage of CD11b positive cells, reduced S phase cells, and increased induction of apoptosis, compared to single agent treatments (Fig 3C–3E).

**Fig 4:**
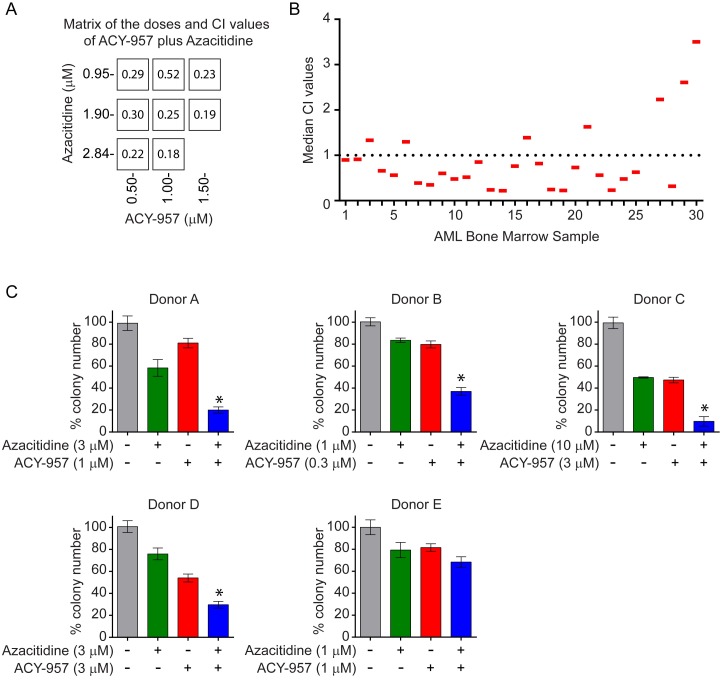
ACY-957 enhances the anti-leukemic activity of azacitidine in primary AML blasts *ex vivo*. (A) Fresh bone marrow samples from AML patients (n = 30) were treated with 3 doses of ACY-957 plus 3 doses of azacitidine. Single agent treatment at 8 doses per drug was performed as well. CI values for 8 combinations were calculated and shown in the squares. The matrix shows one representative bone marrow sample. (B) The median of the 8 CI values was calculated for each sample and plotted. Twenty-two out of 30 samples had a CI value below 1, indicating synergism in majority of the samples tested. (C) Frozen bone marrow samples from AML patients (n = 5) were treated with the indicated doses of ACY-957, azacitidine, ACY-957 plus azacitidine or DMSO and cultured in methylcellulose-based medium for 14 days as in Fig 2G. Colony numbers were quantified and percentage of colonies in DMSO control was plotted. Combination treatment with ACY-957 plus azacitidine significantly reduced colony growth compared to each single agent in 4 out of 5 samples tested. Mean ± SD, n = 3. For all panels, statistical analysis was performed by two-tailed Student’s t-test. * indicates P < 0.05 for all 3 comparisons: combination treatment versus DMSO; combination treatment versus azacitidine alone; combination treatment versus ACY-957 alone.

We next examined the combination of ACY-957 with azacitidine on primary leukemic blasts derived from AML patients in 2D proliferation and 3D colony formation assays. In the 2D proliferation assay, fresh bone marrow samples (n = 30) were subjected to single agent and combination treatment for 96 hours and viable leukemic cells were quantified by flow cytometry as described in Fig 2F. Specifically, eight doses were tested for each single agent. In the combination treatment, 3 doses of azacitidine, 0.9 μM, 1.8 μM and 2.8 μM and 3 doses of ACY-957, 0.5 μM, 1.0 μM and 1.5 μM were tested for each sample and 8 CI values generated (Fig 4A). The median of these 8 values was plotted for each individual sample tested (Fig 4B). Twenty-two out of thirty samples (73.3%) had median CI values below 1, indicating a synergism of this combination on the majority of primary AML patient samples. In the 3D colony formation assay, 5 frozen patient bone marrow samples were subjected to single agent and combination treatment at the indicated doses for 14 days (Fig 4C). Significantly decreased colony growth was observed after combination treatment versus single agent therapy in 4 out of 5 samples tested (80%; Fig 4C). Together, these data indicate ACY-957 synergizes with azacitidine in inhibiting proliferation of primary AML blasts from patients.

### ACY-957 synergizes with azacitidine to induce anti-leukemic activity in MOLM-13 AML xenograft model

**Fig 5:**
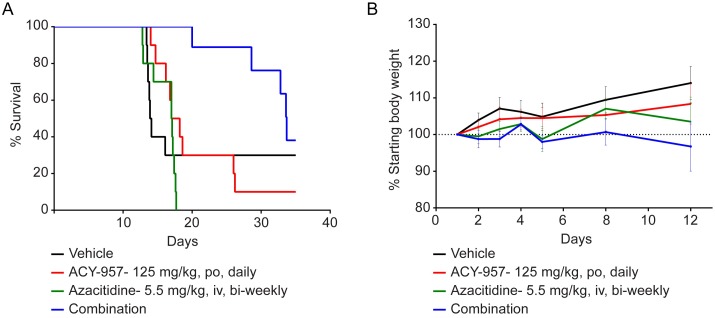
ACY-957 enhances the anti-leukemic activity of azacitidine in MOLM-13 xenograft model. (A-B) Female Crl:NU(NCr)-*Foxn1*^*nu*^ mice implanted with MOLM-13 cells (n = 10/group) were treated with vehicle, ACY-957 (125 mg/kg, po, daily), azacitidine (5.5 mg/kg, iv, bi-weekly), or the combination of ACY-957 plus azacitidine for 28 days. (A) Tumor volume was monitored and TTE for each mouse was calculated and plotted as described in the Materials and Methods. Combination treatment of ACY-957 with azacitidine significantly increased the survival of MOLM-13 model over azacitidine alone (*P* < 0.0001) as well as ACY-957 alone (*P* = 0.005). (B) Body weight was measured on the indicated days and percentage of body weight plotted. Mean ± SD, n = 10. Statistical analysis was performed by Log-rank (Mantel-Cox) test.

To examine the *in vivo* efficacy of ACY-957 in combination with azacitidine, the effect of single agent and combination treatment on survival of the MOLM-13 xenograft model was assessed at the indicated dosing schedules (Fig 5A). Tumors were monitored twice weekly. TTE for each mouse was determined and survival curves plotted as described in the Materials and Methods (S2 Table). The median survival was 14 days in the vehicle control; 17 days in the azacitidine alone group; 17.6 days in the ACY-957 alone group; and 33.7 days in the combination treated group (Fig 5A). At the indicated dosing schedules, combination treatment significantly increased survival in this model over azacitidine alone (*P* < 0.0001) as well as ACY-957 alone (*P* = 0.005). Importantly, this combination treatment was well tolerated in mice and adding ACY-957 to azacitidine did not significantly impact body weight in these animals (Fig 5B). Therefore, ACY-957 synergized with azacitidine to inhibit leukemia tumor growth *in vivo*.

### HDAC1/2 inhibition and azacitidine synergize to drive expression of GATA2 and other AML tumor suppressors

**Fig 6:**
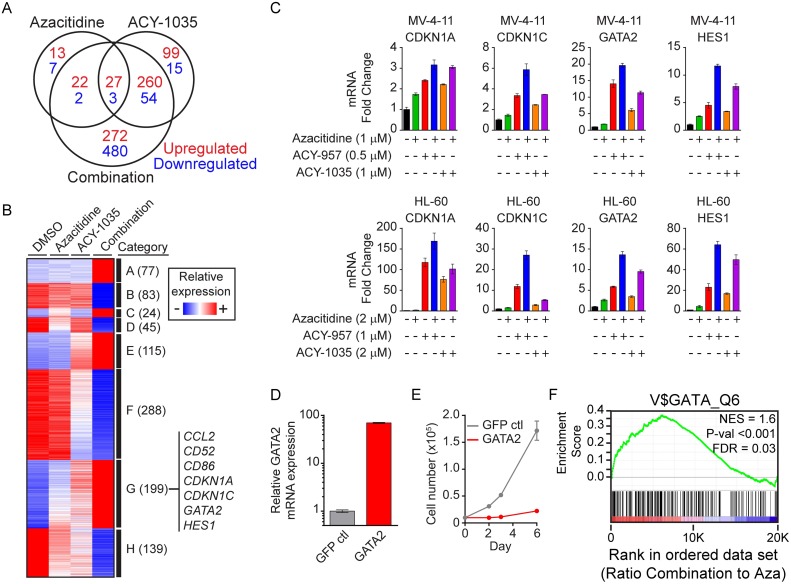
Gene expression profiling identifies differentially regulated genes. (A–B) MV-4-11 cells were treated with vehicle DMSO, azacitidine at 1 μM, ACY-1035 at 1 μM and the combination for 48 hours. Total RNA was prepared and analyzed by Affymetrix GeneChip PrimeView^™^ Human Gene Expression Array. (A) Genes that are up-regulated (red) or down-regulated (blue) by single or combo treatment relative to vehicle. (B) Genes exhibiting additive or synergistic response in the combination treatment relative to each single agent treatment were clustered into 8 categories and represented in a heatmap: A, genes unaffected by single agent treatments and up-regulated in the combination; B, genes unaffected by single agent treatments and down-regulated in the combination; C, genes up-regulated by azacitidine, unaffected by ACY-1035 and up-regulated in the combination more than azacitidine alone; D, genes down-regulated by azacitidine, unaffected by ACY-1035 and down-regulated by the combination more than azacitidine alone; E, genes unaffected by azacitidine, up-regulated by ACY-1035 and up-regulated by the combination more than ACY-1035 alone; F, genes unaffected by azacitidine, down-regulated by ACY-1035 and down-regulated by the combination more than ACY-1035 alone; G, genes up-regulated by azacitidine, up-regulated by ACY-1035 and up-regulated by the combination more than either single agent alone; H, genes down-regulated by azacitidine, down-regulated by ACY-1035 and down-regulated by the combination more than either single agent alone. (C) MV-4-11 and HL-60 cells were treated for 48 hours with the indicated doses of ACY-957, ACY-1035, azacitidine or the combinations. qPCR analysis was performed for the following genes from category G: *CDKN1A*, *CDKN1C*, *GATA2* and *HES1*. *ACTB* was used as the normalizing control. Combinations of ACY-957 plus azacitidine or ACY-1035 plus azacitidine further increased the expression of these genes compared to single agent treatments. Mean ± SD, n = 3. Data shows one representative of 3 independent experiments. (D–E) MV-4-11 cells were transduced with GATA2 or GFP overexpression vectors and stable cell lines derived. (D) Overexpression of GATA2 was confirmed by qPCR. Mean ± SD, n = 3. (E) Stable cells were subjected to proliferation assay in 96-well plates for 6 days. Cell viability was determined at the indicated time points and viable cell numbers plotted. GATA2 overexpressing cells grew much slower than GFP expressing cells. Mean ± SD, n = 3. Data shows one representative of 3 independent experiments. (F) GSEA was performed on the array data using the ‘C3 motif TFT: transcription factor genes’ gene set database. Genes containing *GATA* binding sites within their promoter regions were enriched in the combination of ACY-1035 and azacitidine compared to azacitidine alone. Significant enrichment is illustrated by the positive running enrichment score (ES) marked by the green line, normalized enrichment score (NES) = 1.6, and false discovery rate (FDR) = 0.03.

To explore the molecular mechanisms underlying the combinatorial effects of HDAC1/2 inhibition and azacitidine, RNA transcriptome analysis was performed on MV-4-11 cells treated with vehicle DMSO, azacitidine, ACY-1035 or the combination for 48 hours. Data was analyzed and processed as described in the Materials and Methods and ~ 50,000 probes corresponding to ~ 20,000 genes were analyzed (Fig 6A). Azacitidine alone had only minor effects on gene expression, with 62 genes being up-regulated and 12 genes down-regulated greater than 2-fold relative to vehicle control. ACY-1035 treatment resulted in 386 up-regulated and 72 down-regulated genes using the same criteria. Compared to either single agent, greater numbers of genes were differentially regulated upon combination treatment with 581 up-regulated genes and 539 down-regulated genes. The majority of these genes (752 of 1120 genes) were exclusively changed in the combination group, suggesting a synergistic effect on gene expression by the combination of ACY-1035 with azacitidine. It is conceivable that the differentially regulated genes include genes directly regulated by the treatment through epigenetic modifications at their promoter regions and genes indirectly regulated through additional factors.

Next, differentially regulated genes were clustered into 8 categories based on their relative response to each treatment (Fig 6B and S1 Table). Based on the anti-leukemic activities observed with single agent and combination treatment shown above and the fact that HDAC inhibitors and azacitidine can induce re-expression of certain tumor suppressor genes silenced through epigenetic mechanisms in cancer [62, 65–70], we focused on category G, which includes genes up-regulated by each single agent and further up-regulated by the combination, for further validation. Four genes were selected for validation by qPCR in MV-4-11 and HL-60 lines treated with azacitidine, ACY-957, ACY-1035, ACY-957 plus azacitidine or ACY-1035 plus azacitidine (Fig 6C). *CDKN1A*, a well-known tumor suppressor gene and target of HDAC inhibitors as well as azacitidine, was up-regulated by single agent treatments and further up-regulated by the combinations. Similar observations were made with *CDKN1C*, an established cell cycle regulator with tumor suppressor function, and the transcription factors *GATA2* and *HES1* (Fig 6C).

A recent study demonstrated that the *Gata2* locus is silenced via DNA methylation in AML progenitor cells and the re-expression of Gata2 resulted in significant inhibition of leukemogenesis *in vivo* in an AML mouse model [71]. Consistent with this prior finding, overexpression of GATA2 in MV-4-11 cells led to significant growth inhibition (Fig 6D and 6E). Interestingly, gene set enrichment analysis (GSEA) showed that genes containing *GATA* binding sites within their promoter regions were significantly enriched by the combination of ACY-1035 and azacitidine compared to azacitidine alone, suggesting GATA2 may play a role in mediating the combinatorial effect of HDAC1/2 inhibition and azacitidine (Fig 6F). Together, gene expression profiling identified critical genes involved in the anti-leukemic activities of this drug combination.

## Discussion

Dissection of individual HDACs indicated that specific HDACs control unique cellular functions, providing the rationale for developing isozyme-specific inhibitors to achieve better safety profiles [72–74]. Importantly, it has been reported that HDAC1 and HDAC2 play essential and redundant roles in leukemia cell survival and proliferation, establishing the plausibility that HDAC inhibitors selective for these two isozymes might retain anti-leukemic activity while obviating toxicity associated with inhibition of other HDACs, such as HDAC3 [22, 23, 47, 48, 75]. In fact, HDAC3 plays an essential role in maintaining chromatin structure and genome stability [76, 77]. Mouse knockout studies have also shown that HDAC3 is necessary for maintenance of several metabolic functions in both cardiac and hepatic tissues [78, 79]. In this study we demonstrated that HDAC1/2-selective inhibitors, namely ACY-957 and ACY-1035 of the biaryl aminobenzamide class, inhibited the viability of AML cells, increased their expression of the differentiation marker CD11b, and induced cell cycle arrest and apoptosis, both as single agents and in combination with azacitidine. Analysis of patient bone marrow samples demonstrated that a large majority of these primary AML samples were highly sensitive to the combination of ACY-957 with azacitidine, and ACY-957 significantly prolonged the survival and enhanced the anti-tumor activity of azacitidine in an AML xenograft model without adding additional toxicities. Together, these results demonstrate that selective pharmacological inhibition of HDAC1/2 is sufficient to mediate synergistic anti-cancer activity in combination with azacitidine in preclinical models of AML. These findings have laid a foundation for further investigations to characterize the pharmacology and therapeutic index of this combination in efficacy and toxicology studies. Furthermore, additional studies to explore the combination of HDAC1/2 inhibitors with a range of existing and emerging AML therapies are warranted.

DNA methylation and histone acetylation are well-characterized epigenetic mechanisms which can result in repression of tumor suppressor genes and cell transformation [80] [62, 68, 69]. In fact, reduction of DNA methylation by azacitidine and induction of histone acetylation by HDAC inhibitors, both of which lead to reactivation of tumor suppressor genes, are believed to contribute significantly to their therapeutic effects. Gene expression profiling in this study identified critical AML tumor suppressor genes which may play important roles in mediating the combinatorial effects of HDAC1/2 inhibition with azacitidine. Specifically, gene expression profiling showed that 199 genes were further up-regulated by combination treatment over either single agent, among which several tumor suppressor genes were identified. CDKN1A (p21) and CDKN1C (p57) are cyclin-dependent kinase inhibitors of several G1 cyclin complexes [81–83]. Overexpression of CDKN1A or CDKN1C can arrest cells at G1 phase [82, 83]. Therefore, these genes may play important roles in mediating the suppressive effects of the combination treatment on cell cycle progression. HES1 and GATA2 are transcription factors that may play important roles in driving the broad changes in gene expression resulting from combination treatment. Elevated endogenous levels of HES1 have been associated with longer relapse-free survival and increased overall survival in AML patients [84], and overexpression of HES1 in AML cell lines suppressed their growth and increased the expression of CDKN1A [85, 86]. GATA2 is known to be dysregulated through genetic mutations or epigenetic silencing in subsets of AML and myelodysplastic syndrome [71, 87–92]. GATA2 was shown to be hypermethylated in *TET2;Flt3*^ITD^ AML progenitor cells and ectopic expression of GATA2 significantly reduced the tumorigenicity of these cells [71]. Moreover, GATA2 has been shown to be a direct target of ACY-957 in hematopoietic progenitor cells [52]. In agreement with these results, we also found that GATA2 expression was induced in AML cells by HDAC1/2 inhibitors or azacitidine and this induction was further enhanced by combination treatments. Furthermore, ectopic expression of GATA2 reduced the proliferation of MV-4-11 cells, suggesting GATA2 might play a role in mediating the anti-leukemic activity of the combination of HDAC1/2 inhibition with azacitidine. In addition, our analysis identified additional 7 categories of genes which exhibited additive or synergistic changes in the combination treatment relative to single agent treatments (Fig 6B). It is possible that some of these genes can contribute to the anti-leukemic activities of this combination as well.

Together, our findings demonstrate that selective HDAC1/2 inhibitors are efficacious both as single agents and in combination with azacitidine in inhibiting proliferation of AML cell lines and primary AML blasts from patients. Importantly, HDAC1/2 inhibitor ACY-957 significantly enhances the anti-leukemic activity of azacitidine in the MOLM13 xenograft model of AML. Further preclinical studies are warranted to characterize the therapeutic application of HDAC1/2 inhibitors in AML in multiple combination settings.

## Supporting Information

S1 Fig ACY-1035 did not inhibit HDAC4 through 9 at the maximum concentration tested of 20 μM. (PDF)

S2 Fig HDAC1/2 inhibition increases histone acetylation. (PDF)

S3 Fig HDAC1/2 inhibition reduces viability of AML cells *in vitro*. (PDF)

S4 Fig ACY-957 inhibits proliferation of primary leukemic blasts from AML patients. (PDF)

S1 Table Gene expression profiling analysis. (XLSX)

S2 Table TTE for individual mouse. (XLSX)
