# Supplementary material for: Selective Inhibitors of Histone Deacetylases 1 and 2 Synergize with Azacitidine in Acute Myeloid Leukemia
Source: PLoS One. 2017 Jan 6;12(1):e0169128. doi: 10.1371/journal.pone.0169128 (PMC5218480; doi:10.1371/journal.pone.0169128)
Supplement: S1 Fig — (PDF) [file pone.0169128.s001.pdf]

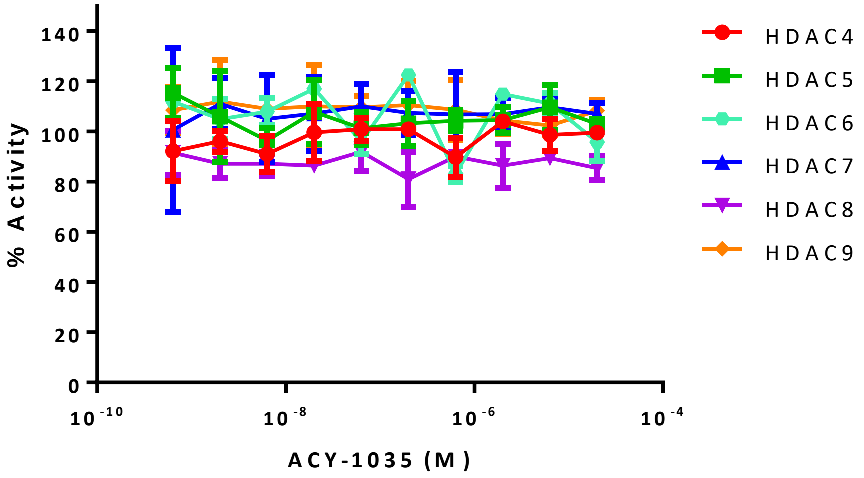

S1 Fig. ACY-1035 did not inhibit HDACs 4 through 9 at the maximum concentration tested of 20 μM. *In vitro* biochemical assay for HDAC4, HDAC5, HDAC6, HDAC7, HDAC8, HDAC9 inhibition by ACY-1035.
