# Supplementary material for: Selective Inhibitors of Histone Deacetylases 1 and 2 Synergize with Azacitidine in Acute Myeloid Leukemia
Source: PLoS One. 2017 Jan 6;12(1):e0169128. doi: 10.1371/journal.pone.0169128 (PMC5218480; doi:10.1371/journal.pone.0169128)
Supplement: S2 Fig — (PDF) [file pone.0169128.s002.pdf]

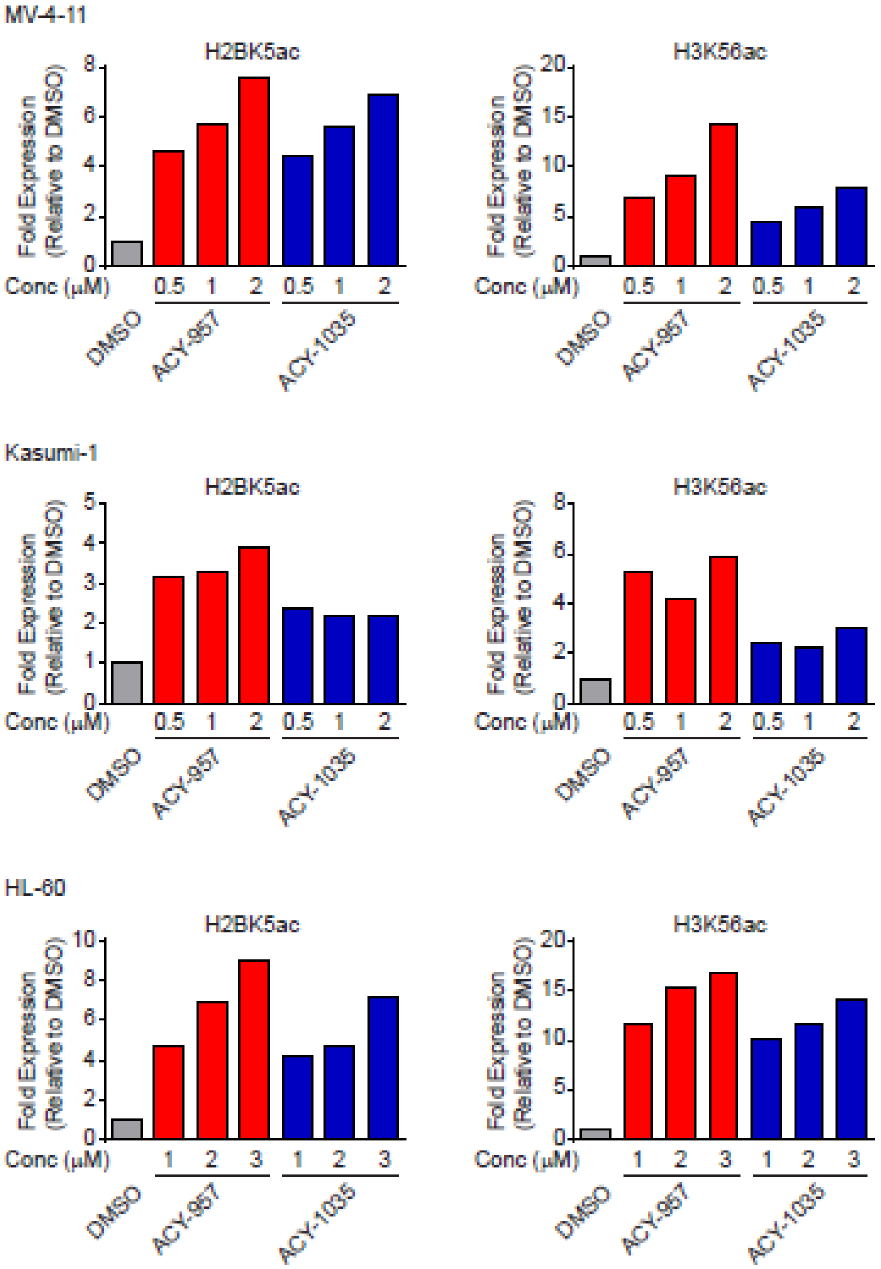

S2 Fig. HDAC1/2 inhibition increases histone acetylation. MV-4-11, Kasumi-1 and HL-60 cells were treated at the indicated doses of compounds for 24 hours and immunoblotting performed for acetylated H2BK5, acetylated H3K56 and total H4 as in Fig 1B. The relative abundance of each acetylated histone mark relative to total histone H4 was normalized to DMSO and plotted.
