# Supplementary material for: Selective Inhibitors of Histone Deacetylases 1 and 2 Synergize with Azacitidine in Acute Myeloid Leukemia
Source: PLoS One. 2017 Jan 6;12(1):e0169128. doi: 10.1371/journal.pone.0169128 (PMC5218480; doi:10.1371/journal.pone.0169128)
Supplement: S3 Fig — (PDF) [file pone.0169128.s003.pdf]

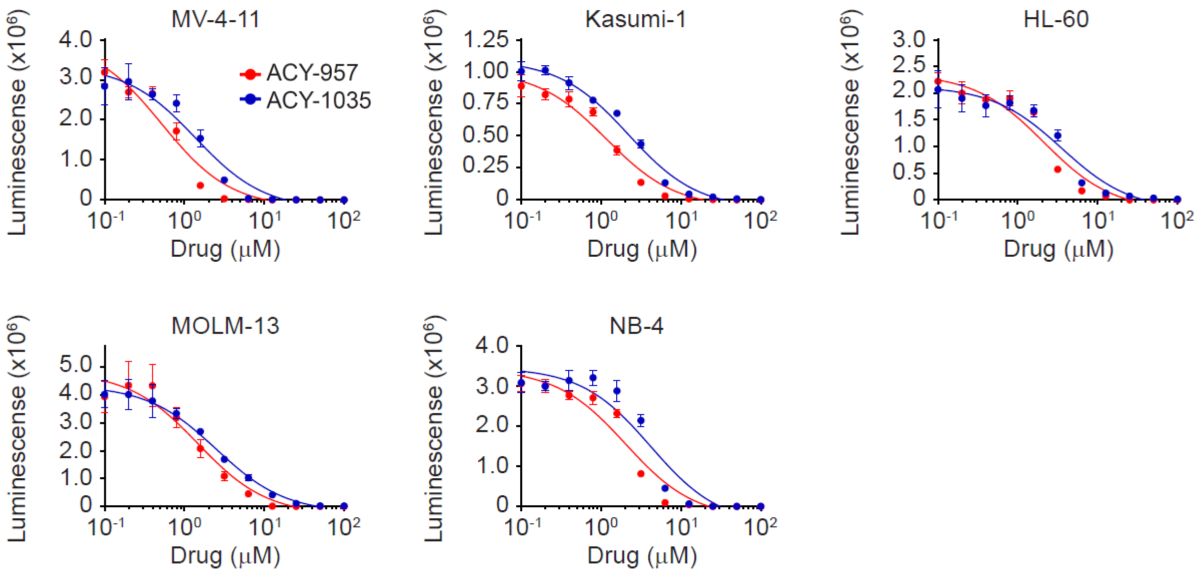

S3 Fig. HDAC1/2 inhibition reduces viability of AML cells *in vitro*. MV-4-11, Kasumi-1, HL-60, MOLM-13 and NB-4 cells were treated with increasing doses of ACY-957 or ACY-1035. After 72 hours, cell viability was measured and response curves plotted using GraphPad Prism 6.
