# Supplementary material for: Selective Inhibitors of Histone Deacetylases 1 and 2 Synergize with Azacitidine in Acute Myeloid Leukemia
Source: PLoS One. 2017 Jan 6;12(1):e0169128. doi: 10.1371/journal.pone.0169128 (PMC5218480; doi:10.1371/journal.pone.0169128)
Supplement: S4 Fig — (PDF) [file pone.0169128.s004.pdf]

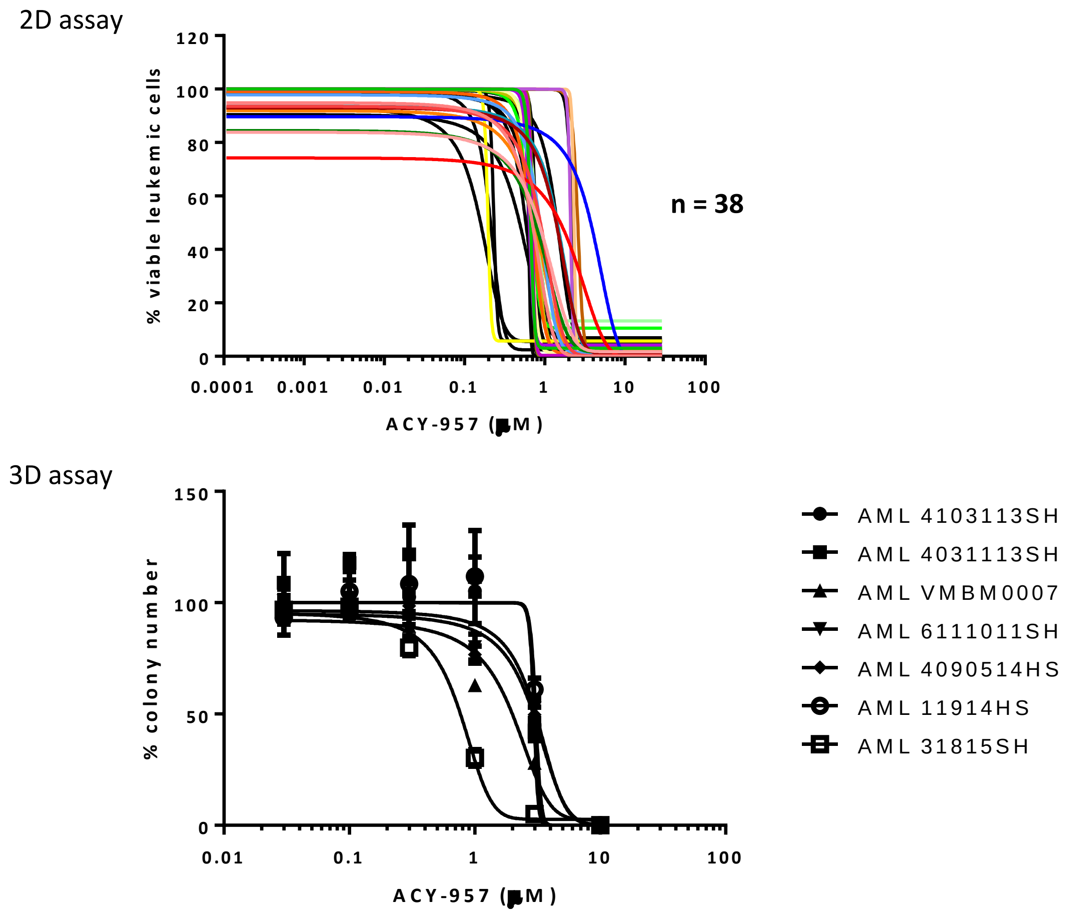

S4 Fig. ACY-957 inhibits proliferation of primary leukemic blasts from AML patients. For 2D assay, fresh primary AML patient samples (n = 38) were plated into 96-well plates containing ACY-957 at 8 doses and incubated at 37°C for 96 hours. Viable leukemic cells were quantified by flow cytometry and the response curves were plotted using GraphPad Prism 6. For 3D assay, frozen primary AML patient samples (n = 7) were cultured in methylcellulose-based medium and treated with 6 doses of ACY-957 for 14 days. Colonies were quantified and percentage of colony number relative to DMSO treated control was plotted.
